# Oral mycobiome composition and fungi-bacteria associations in children and young adults with type 1 diabetes

**DOI:** 10.1080/20002297.2026.2750003

**Published:** 2026-10-29

**Authors:** Giuseppina Campisciano, Luana Aldegheri, Nunzia Zanotta, Eulalia Catamo, Antonietta Robino, Francesca Mione, Milena Cadenaro, Chiara Navarra, Gianluca Tornese, Manola Comar

**Affiliations:** a Institute for Maternal and Child Health - IRCCS ‘Burlo Garofolo’, Trieste, Italy; b Department of Medicine, Surgery and Health Sciences, University of Trieste, Trieste, Italy

**Keywords:** Oral mycobiome, type 1 diabetes, fungi–bacteria associations, children, young adults, oral microbiome

## Abstract

**Background:**

Oral mycobiome in children and young adults with type 1 diabetes (T1D) remains poorly characterized.

**Objective:**

We investigated oral fungal community structure and its associations with bacterial communities and immune mediators in individuals with established T1D compared with healthy controls (HC).

**Design:**

Oral fungal and bacterial communities were characterized in 68 participants with T1D and 69 HC by ITS and 16S rRNA gene sequencing, alongside multiplex assessment of soluble immune factors. Sequencing data were processed with QIIME 2, immune factors with BioPlex Manager and statistical analyzes with R v.4.3.3.

**Results:**

Fungal alpha and beta diversity did not differ significantly between groups. However, several taxa were differentially abundant or prevalent in T1D, including *Fusarium, Hanseniaspora, Meyerozyma, Cyberlindnera jadinii, Candida albicans, C. dubliniensis* and *Malassezia globosa*. No significant differences emerged by HbA1c category (<7% *vs*. ≥7%) within T1D. Notably, *Cystobasidium/C. slooffiae* and *Trichoderma*, detected exclusively in T1D, showed stronger positive associations with MCP-1/MCAF and IP-10, respectively, than in HC, while several fungal genera were inversely associated with distinct bacterial taxa.

**Conclusions:**

These findings indicate that T1D is associated with selective, taxon-specific oral mycobiome alterations rather than generalized dysbiosis. The observed fungal-immune and fungal-–bacterial links suggest biological relevance, warranting longitudinal and functional investigation.

## Introduction

Type 1 diabetes (T1D) is a chronic autoimmune disorder characterized by immune-mediated destruction of pancreatic *β*-cells, resulting in lifelong insulin dependence and persistent hyperglycemia. Although T1D can occur at any age, it most commonly develops in children and adolescents [1].

T1D results from a complex interplay of both genetic and environmental factors [2]. Increasing evidence suggests a possible role of alterations in both gut and oral microbiota, often characterized by reduced microbial diversity and an increased abundance of bacterial genera associated with inflammation and opportunistic behavior [3]. Moreover, oral dysbiosis may also influence gut microbial communities, suggesting a potential interplay between these two microbial sites that may contribute to the inflammatory processes involved in T1D [4].

The oral cavity hosts the second most complex and diverse microbial ecosystem in the human body, after the gut, contributing to the maintenance of both local and systemic health [5]. In our previous study, reduced bacterial alpha diversity in subjects with T1D were already observed, alongside enrichment of pathobionts such as *Atopobium* and *Prevotella* and depletion of beneficial taxa. These shifts correlated with an altered inflammatory profile, marked by significantly elevated IL(Interleukin)-1β levels in T1D compared to healthy controls [4].

While the bacterial component of the oral microbiota has been extensively studied, the fungal community, known as the oral mycobiome, remains largely unexplored. Although fungi account for a smaller proportion of the oral microbial ecosystem, they may play an important role through interactions with bacterial communities, modulation of the host's immune response and contribution to both oral homeostasis and disease [6].

In healthy individuals, the oral mycobiome is generally characterized by a relatively stable community, composed of around 100 fungal species, with the more frequent and abundant component described as the ‘*core mycobiome*’ [7]. The most detected genus is *Candida*, followed by *Cladosporium*, *Saccharomyces*, *Aspergillus* and *Cryptococcus* [8,9].

As for bacteria, alterations in fungal composition have been associated with pathological conditions, including dental caries, periodontal disease and systemic disorders [7,10]. However, the contribution of fungi in the development and progression of these disorders remains relatively unexplored, largely due to the limited cultivability of many fungal species using conventional methods and their complex genetic characteristics [11].

Results from studies employing next-generation sequencing techniques suggest that fungal and bacterial gut dysbiosis, together with intestinal inflammation, is associated with the development of T1D. Certain fungal genera may modulate host immune responses and inflammatory pathways, potentially contributing to the altered local and systemic inflammatory state characteristic of T1D [7,12]. Overall, these findings highlight the oral fungi-bacteria interkingdom network as a promising area of investigation for better characterizing microbial associations in T1D, including their potential relationship with the inflammatory profile.

Therefore, the main aim of this study was to determine whether established T1D was associated with alterations in the oral mycobiome of children and young adults compared with healthy controls, using saliva as a validated proxy for the oral microbiome. Secondly, we examined the clinical, inflammatory and bacterial correlates of fungal composition, including glycemic control, immune mediators and fungal-bacterial associations.

## Materials and methods

### Study cohort

In the present study, 68 subjects diagnosed with T1D and 69 healthy controls (HC) were recruited from the Diabetes Unit and the Emergency Department of IRCCS Burlo Garofolo (Trieste, Italy). Inclusion criteria for participants with T1D were: diagnosis of T1D for at least 1 year, age between 6 and 21 years and absence of other types of diabetes mellitus (i.e. type 2, monogenic diabetes, cystic fibrosis-related diabetes). Inclusion criteria for HC were: age between 6 and 21 years, no diagnosis of T1D or any other diabetes forms, absence of obesity or other metabolic disorders, HbA1c ≤ 6% (≤42 mmol/mol) and no family history of diabetes. To minimize selection bias, for all participants, we collected detailed information on their primary medical conditions and oral health status through two complementary sources: a standardized pediatric dental examination performed by a trained dentist, and a structured questionnaire completed by the participants and/or their parents/legal guardians at the time of enrollment. These data included information on current acute illnesses (e.g. fever, respiratory or gastrointestinal infections), chronic inflammatory or systemic conditions, ongoing medications and oral health problems (e.g. dental caries, gingivitis, periodontal disease, oral mucosal lesions). Based on these assessments, we applied predefined exclusion criteria to minimize the inclusion of patients with active inflammatory or oral diseases that could confound the oral microbiome profile. Controls were recruited among patients attending the Emergency Department for minor, non‑infectious and non‑chronic conditions (e.g. minor traumas, sprains, superficial wounds).

The exclusion criteria for all participants were the use of antimycotics, antibiotics or anti-inflammatory drugs within 14 days prior to sampling.

Age, sex and HbA1c were collected at the time of enrollment. HbA1c was measured with finger pricks using portable instrumentation at outpatient clinics (QuikRead go, A. De Mori S.p.A., Milan, Italy). Subjects with T1D were stratified by median HbA1c over the past year: ≥7% (suboptimal glycemic control) *vs.* <7% (good glycemic control), based on the ISPAD Clinical Practice Consensus Guidelines 2022, which recommend a target of <53 mmol/mol (<7%) for all young people with diabetes to reduce the risk of long‐term microvascular and macrovascular complications [13]. All T1D participants were on insulin therapy, administered either through multiple daily injections or continuous subcutaneous insulin infusion. The research project was approved by the Ethic Committee (CEUR-2018-Em-323-Burlo). Before enrollment, all participants or their parents/guardians (for participants aged < 18 years) provided written informed consent.

### Saliva collection and DNA extraction

Each participant was asked to collect 2 mL of saliva and, upon arrival at the laboratory, samples were stored at −80 °C until further processing. Subjects were required to avoid brushing their teeth with fluoride toothpaste for 12 hours prior to sample collection to avoid its antifungal activity [14] and to avoid sugar-sweetened beverages for at least 1 hour before saliva collection. The clinical periodontal parameters, specifically bleeding on probing (BoP%) and plaque index (PI%), were assessed at the site level, with mean values calculated for each subject.

For DNA extraction, 300 μL of each sample was used, with a final elution volume of 50 μL, using the Maxwell CSC instrument (Promega Srl, Madison, WI, USA) in accordance with the manufacturer's instructions. The concentration (ng/μL) of the nucleic acids was assessed by the NanoDrop® 1000 spectrophotometer (Thermo Fisher Scientific, Waltham, MA, USA).

### ITS and 16S rRNA genes targeted sequencing

The fungal composition of the samples was profiled through Internal Transcribed Spacer (ITS) region-targeted sequencing, whereas the bacterial composition by the V3-V4 regions of the 16S rRNA gene sequencing. Library preparation was performed using the Quick-ITS Plus NGS Library Prep Kit and the Quick-16S Plus NGS Library Prep Kit (V3-V4, UDI) (Zymo Research, Irvine, California, USA), respectively. The ITS2 region (~480 bp) of the ITS region was amplified using primers ITS3f (GCATCGATGAAGAACGCAG) and ITS4r (TCCTCCGCTTATTGATATGC), while the V3-V4 regions (~606 bp) of the 16S rRNA gene was amplified using 341f (mixture of CCTACGGGDGGCWGCAG and CCTAYGGGGYGCWGCAG primers) and 806r (GACTACNVGGGTMTCTAATCC) primers. An equal amount of each amplified product was mixed into a single batch to generate the ITS region pooled library and the 16S rRNA pooled library, according to the manufacturer's instructions.

Purification of the obtained pooled libraries was achieved using the Select-a-Size DNA Clean & Concentrator MagBead Kit (Zymo Research, Irvine, California, USA) and quantification was performed using a Qubit® 2.0 Fluorimeter (Invitrogen, Carlsbad, CA, USA) in combination with the Qubit® dsDNA HS Assay Kit (Thermo Fisher Scientific, Waltham, MA, USA).

For the NextSeq® Reagent Kit P1/P2 (600-cycle), a final library loading concentration of 750 pM was obtained using onboard denaturation on the Illumina NextSeq1000 platform (Illumina, San Diego, CA, USA) and diluted with 40% PhiX spike-in. A no-template control was processed and sequenced together with the biological samples.

QIIME 2 software v2024.5 (https://qiime2.org) was used for raw sequence data processing. Denoizing and chimera removal were performed with DADA2 implemented in QIIME2, and high-quality reads (Q ≥30) were retained. The negative control did not yield high-quality reads after DADA2 filtering, suggesting the absence of detectable contaminant DNA.

Fungal taxonomic classification was carried out using the UNITE reference database v10.0, while bacterial taxonomic classification was achieved by the SILVA reference database v138. To handle variability in sequencing read depth, bacterial abundance data were normalized using Total Sum Scaling (TSS).

### Immunoassay

A panel of 27 cytokines, chemokines and growth factors (BioPlex Pro™ human cytokine 27-plex panel; Bio-Rad Laboratories, Milan, Italy) were dosed in saliva using magnetic bead-based multiplex immunoassays, according to the manufacturer's instructions.

The concentrations of the tested factors were determined using the Luminex 200 system (Bio-Rad Corp., Hercules, CA, United States) and BioPlex Manager software (v.6; Bio-Rad). Results were reported as concentration (pg/mL).

### Statistical analysis

The statistical analysis was designed to assess whether type 1 diabetes (T1D) was associated with alterations in the oral mycobiome and to explore potential clinical, inflammatory and bacterial correlates of fungal community composition. The primary comparison was between participants with T1D and healthy controls (HC). Secondary analyzes investigated whether fungal diversity and composition were associated with glycemic control within the T1D group, with HbA1c categorized as <7% *vs*. ≥7%, and whether fungal taxa were associated with inflammatory mediators and bacterial taxa.

Demographic and periodontal characteristics were compared between T1D and HC groups to assess baseline comparability and to identify clinical variables potentially relevant to the interpretation of mycobiome differences. Age was assessed as a continuous variable and sex as a categorical variable. Periodontal parameters included bleeding on probing (BoP%) and plaque index (PI%).

The distribution of continuous variables was assessed for normality. Differences in age were evaluated using an independent-samples t-test, whereas sex distribution was compared using the chi-square test of independence. BoP% and PI% were compared using the Mann-Whitney U test because of their non-normal distribution.

HbA1c was considered a marker of glycemic control and was evaluated within the T1D group. For secondary analyzes, participants with T1D were stratified according to HbA1c < 7% *vs*. ≥7%.

Age, sex, BoP% and PI% were considered a priori as potential confounding variables because of their potential influence on oral microbial composition. Their associations with fungal taxa were examined using multivariable MaAsLin2 models. These analyzes were used to assess whether these clinical and demographic variables were associated with fungal composition and to inform their inclusion as covariates in the main multivariable analyzes.

Importantly, these variables were treated as potential covariates rather than as primary exposures of interest. All multivariable models evaluating the association between T1D status and fungal composition were adjusted for age, sex, BoP% and PI%, where applicable.

Alpha diversity was used to characterize within-sample fungal diversity. Observed amplicon sequence variants (ASVs), Shannon diversity and Simpson diversity indices were calculated. Differences in alpha diversity between T1D and HC were evaluated using linear models, with T1D status as the primary predictor and age, sex, BoP% and PI% included as covariates in multivariable models.

Alpha diversity was also evaluated within the T1D group according to HbA1c category (<7% *vs*. ≥7%) to investigate whether glycemic control was associated with fungal diversity.

Beta diversity was assessed using Bray-Curtis dissimilarity based on fungal relative abundance profiles. Principal coordinates analysis (PCoA) was used to visualize the overall similarity of fungal communities among samples. Differences in community composition between T1D and HC were formally assessed using permutational multivariate analysis of variance (PERMANOVA; adonis2) with 999 permutations, adjusted for age, sex, BoP% and PI%. The proportion of variance explained by group status was expressed as R^2^. Homogeneity of multivariate dispersions between groups was assessed using betadisper, with 999 permutations, to determine whether differences in dispersion could account for the PERMANOVA results.

The same beta-diversity framework was applied to the comparison of T1D participants according to HbA1c category.

Differences in fungal taxonomic composition between T1D and HC were assessed at the genus and species levels using ANCOM-BC. The analysis was performed to identify individual fungal taxa whose relative abundance differed between the two study groups while accounting for the compositional nature of microbiome data. Multivariable analyzes included age, sex, BoP% and PI% as covariates.

Differential abundance was also evaluated within the T1D group according to HbA1c category to investigate whether fungal taxa were associated with glycemic control.

Statistical significance was assessed using false discovery rate (FDR)-adjusted *p*-values, with q-value < 0.05 considered statistically significant.

Fungal prevalence was defined as the proportion of samples in which a given taxon was detected. Unadjusted differences in prevalence between T1D and HC were assessed using chi-square tests. Multiple comparisons were accounted for using the Benjamini-Hochberg false discovery rate (BH-FDR) correction, with an FDR-adjusted *p*-value < 0.05 considered statistically significant.

Associations between fungal taxa and salivary immune mediators were investigated using multivariable MaAsLin2 models. Cytokine concentrations were treated as continuous predictors, whereas fungal taxa were used as microbial outcomes. Models were adjusted for age, sex, BoP% and PI% to account for potential demographic and periodontal confounding.

MaAsLin2 models were used to assess whether fungal taxon-immune mediator associations differed according to T1D status or glycemic control. For each fungal feature, linear models included the cytokine concentration, the grouping variable (i.e. controls *vs*. individuals with T1D, or HbA1c < 7% *vs*. ≥7% among individuals with T1D), their interaction and the following covariates: age, sex, PI% and BoP%. The interaction term was used to assess whether cytokine-fungal taxon associations differed between groups.

As a descriptive secondary analysis, immune-mediator concentrations were compared between individuals with T1D and HC using the Wilcoxon rank-sum test.

Potential cross-kingdom relationships between bacterial and fungal communities were then investigated. Taxa detected in fewer than 10% of samples were excluded from analyzes based on continuous relative-abundance data. Relative abundances were centered log-ratio (CLR)-transformed to account for the compositional nature of microbiome data. Associations between bacterial and fungal relative abundances, and between bacterial and fungal alpha-diversity measures, were assessed using Spearman's rank correlation coefficient. Relationships based on taxon presence or absence were evaluated using the phi coefficient.

Multiple testing was controlled using the Benjamini‒Hochberg procedure, and FDR-adjusted *p*-values < 0.05 were considered statistically significant. Unless otherwise specified, all tests were two-sided. Analyzes were performed in R version 4.3.3.

## Results

### Study cohort

The study included 68 participants with T1D (48.5% males) with a mean age (±SD) of 14.5 ± 3.3 years, and an HC group of 69 subjects (48% males) with a mean age (±SD) of 13.7 ± 4.5 years. No significant differences in age and sex were observed between T1D and HC, indicating comparable demographic characteristics between the two groups. HbA1c values were significantly higher in participants with T1D than in HC (7.49 ± 1.19% *vs*. 5.26 ± 0.34%, *p*-value < 0.001). Concerning clinical periodontal parameters, BoP% did not significantly differ between T1D and HC, whereas PI% was significantly different (Table 1).

**Table 1.** Demographic characteristics of study participants.

	T1D (*n* = 68)	HC (*n* = 69)	*p*-value
Age, years (mean ± SD)	14.5 ± 3.3	13.7 ± 4.5	0.25
Sex, *n* (% male)	33 (48.5)	33 (48)	0.93
HbA1c %, mean ± SD	7.49 ± 1.19	5.26 ± 0.34	<10^−3^
BoP%, mean ± SD	15.1 ± 14.5	13.8 ± 14.2	0.17
PI%, mean ± SD	49.9 ± 21.1	41.35 ± 26.7	0.02

Demographic and clinical characteristics were compared between T1D and HC groups (t-test for age; χ^2^ for sex; Mann-Whitney U for BoP% and PI%). T1D = type 1 diabetes; HC = healthy controls; BoP = bleeding on probing; PI = plaque index.

### Oral fungal community diversity

After filtering of Viridiplantae, Metazoa and unassigned reads, the sequencing output for the fungal population yielded a total of 3,897,530 sequences (median = 18,497 and IQR = 40,685).

Alpha diversity was assessed using Observed ASVs, Shannon and Simpson indices at phylum, genus and species levels. No significant differences in fungal alpha diversity were observed between T1D and HC for any of the three indices (Table 2), after adjustment for age, sex, BoP% and PI%.

Within the T1D group, no significant differences in alpha diversity were observed between participants with HbA1c < 7% and those with HbA1c ≥7% (Table 2).

**Table 2.** Alpha diversity at the genus level in T1D and HC groups. Alpha diversity was compared between individuals with type 1 diabetes (T1D) and healthy controls (HC), and among individuals with T1D stratified according to HbA1c levels, with HbA1c < 7% indicating good glycemic control. Comparisons were performed using linear models with T1D status as the primary predictor and age, sex, BoP% and PI% included as covariates in multivariable models.

Metric (mean ± SD)	HC	T1D	FDR-adjusted *p*-values	T1D (HbA1c < 7%)	T1D (HbA1c ≥7%)	FDR-adjusted *p*-values
Observed ASVs	10.1 ± 3.3	13.3 ± 6.1	0.06	14.8 ± 6.87	12.4 ± 5.42	0.91
Shannon	0.94 ± 0.56	1.15 ± 0.6	0.48	1.27 ± 0.66	1.06 ± 0.55	0.91
Simpson	0.46 ± 0.26	0.53 ± 0.23	0.44	0.56 ± 0.24	0.51 ± 0.23	0.91

Beta diversity was assessed using Bray-Curtis dissimilarity at the phylum, genus and species levels (Figure 1). No significant differences in fungal community composition between T1D and HC were detected at any of the taxonomic levels investigated. At the genus level, shown in Figure 1A, the PCoA did not show a clear separation of fungal communities by T1D status, and PERMANOVA did not identify a significant difference in overall community composition (R^2^ = 0.009, pseudo-F = 1.12, *p*-value = 0.28). However, assessment of multivariate homogeneity of group dispersions using betadisper revealed significant differences in dispersion between T1D and HC at the genus and species levels (*p*-value = 0.013 and *p*-value = 0.045, respectively), indicating differences in within-group variability rather than in overall community composition. Within the T1D group, no significant differences in beta diversity according to HbA1c category were observed at the phylum, genus and species levels. Figure 1B shows the PCoA at the genus level (R^2^ = 0.032, pseudo-F = 1.57, *p*-value = 0.11).

**Figure 1.**
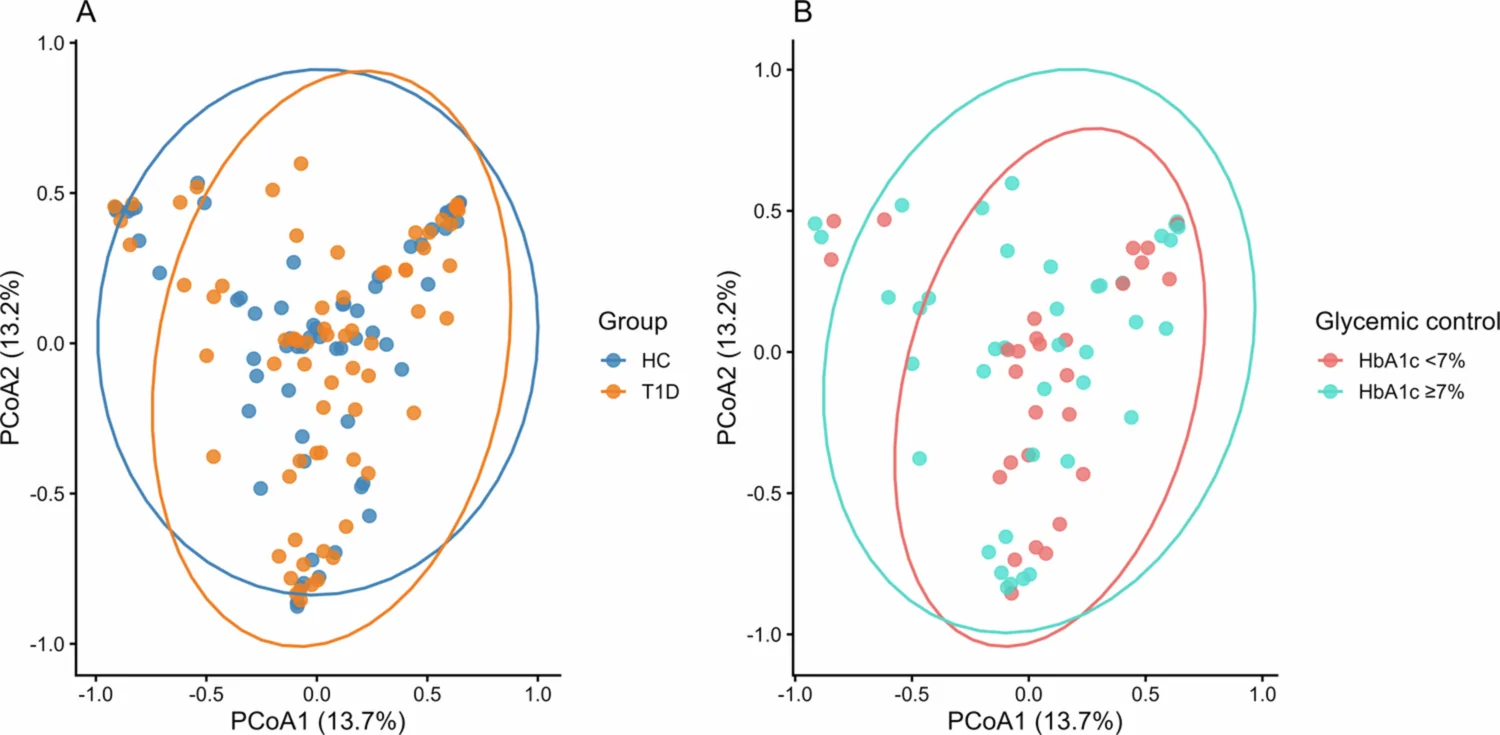
Principal coordinates analysis (PCoA) at genus level based on Bray-Curtis dissimilarity. (A) Fungal community composition in individuals with type 1 diabetes (T1D) and healthy controls (HC). (B) Fungal community composition among individuals with T1D stratified according to glycemic control, with HbA1c < 7% indicating good glycemic control and HbA1c ≥7% indicating suboptimal glycemic control. Differences in community composition were assessed using PERMANOVA (adonis2, 999 permutations), with adjustment for age, sex, BoP% and PI%. Homogeneity of group dispersions was assessed using betadisper (999 permutations).

Although no significant differences were observed in overall fungal alpha- and beta-diversity, an exploratory overview of mean relative-abundance profiles showed differences in the distribution of the most abundant bacterial and fungal genera between HC and T1D (Figure 2). For the bacterial component (Figure 2A), *Prevotella* represented the most abundant genus in both groups, with a higher mean relative abundance in T1D, whereas *Neisseria* showed the opposite pattern, with higher mean abundance in HC. *Streptococcus* and *Veillonella* displayed relatively similar abundances between groups, while more modest differences were observed for *Haemophilus, Porphyromonas, Fusobacterium* and other genera.

For the fungal component (Figure 2B), *Malassezia* was the most abundant genus in both groups, with broadly comparable mean relative abundances. *Saccharomyces* showed a higher mean abundance in T1D, whereas *Candida* and *Cladosporium* appeared more abundant in HC. Several genera occurring at lower mean abundances, including *Cyberlindnera, Hanseniaspora, Meyerozyma, Fusarium, Aspergillus* and *Geotrichum*, showed a tendency toward higher values in T1D. Overall, these patterns provide an exploratory overview of differences in microbial composition between the two groups, without implying statistical significance or differential abundance.

**Figure 2.**
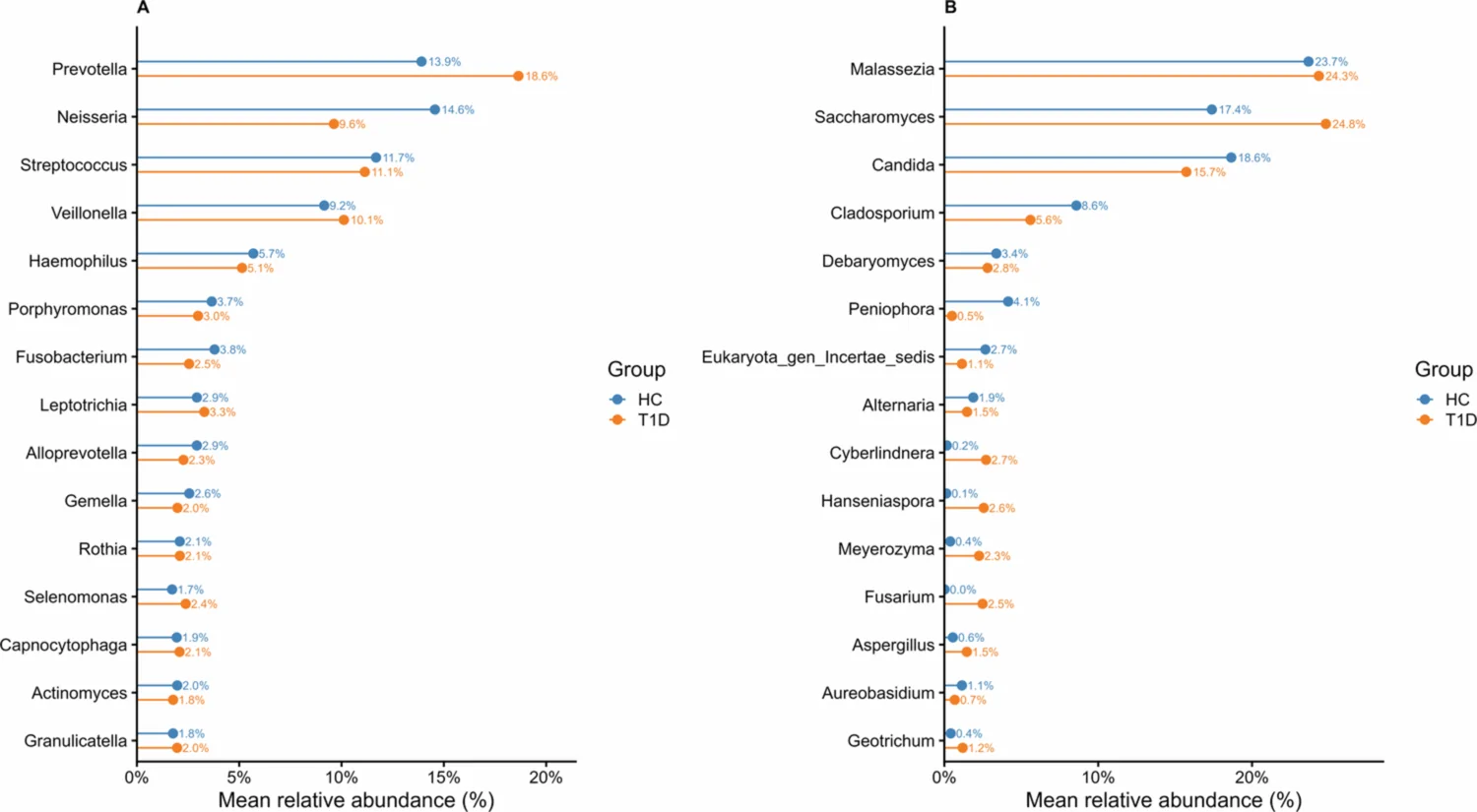
Exploratory overview of mean relative abundances of the predominant bacterial and fungal taxa in healthy controls and patients with type 1 diabetes. Panel A shows the bacterial taxa, whereas panel B shows the fungal taxa. Points represent the mean relative abundance of each taxon in the healthy control group (HC) and the type 1 diabetes group (T1D). The figure is intended for exploratory visualization; statistical significance was not inferred from the plotted values alone.

Differential abundance analysis identified specific fungal taxa associated with T1D status. The ANCOM-BC test identified several differentially abundant taxa: in the T1D group, *Zygosaccharomyces* (q-value = 0.025) was decreased, whereas *Fusarium* (q-value = 3.7 × 10^−5^ *), Hanseniaspora* (q-value = 1.8 × 10^−4^)*, Vuilleminia* (q-value = 5.6 × 10^−3^), *Meyerozyma* (q-value = 0.03), *Cyberlindnera jadinii* (q-value = 4.6 × 10^−7^), *Candida albicans* (q-value = 0.043), *Candida dubliniensis* (q-value = 0.043*)* and *Malassezia globosa* (q-value = 0.043) were increased compared to HC (Figure 3).

**Figure 3.**
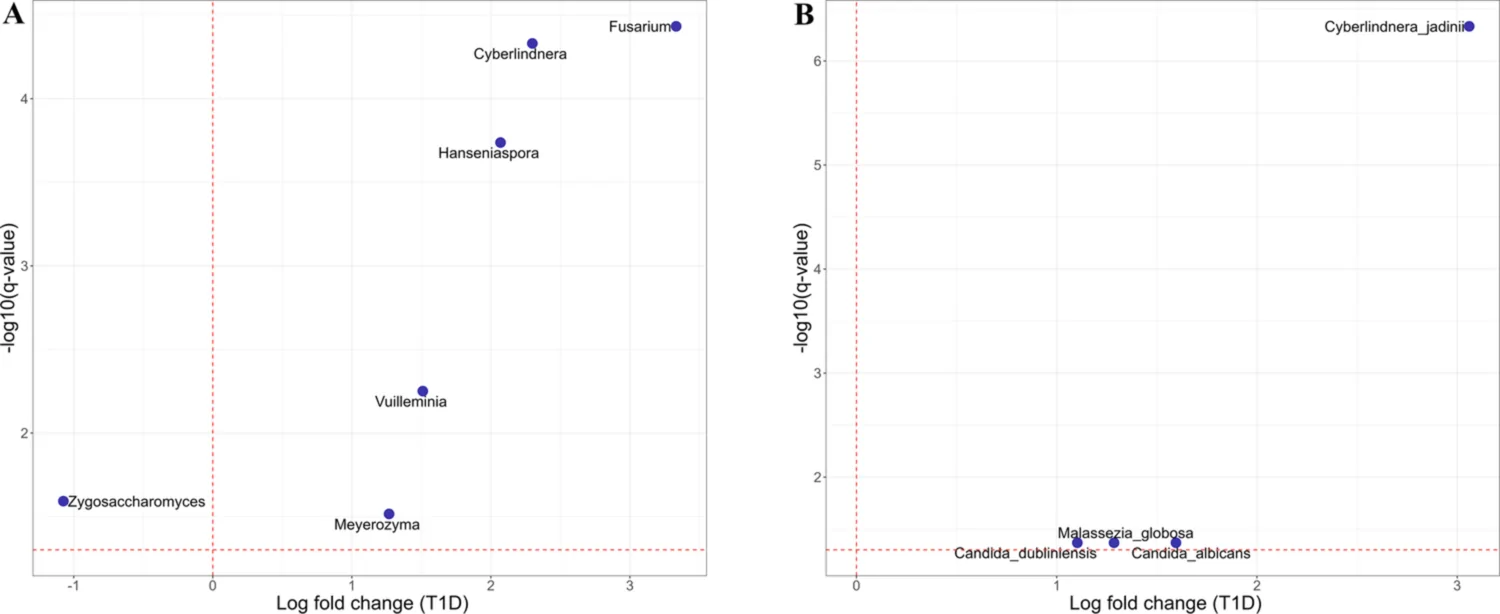
Differential fungal taxa. Volcano plot of significantly modulated fungi at genus (A) and species (B) levels between subjects with type 1 diabetes (T1D) and healthy controls (HC), identified using the ANCOM-BC test adjusted for age, sex, BoP% and PI%, with FDR-adjusted *p*-values (significance threshold: -log10(q-value) > 1.3, corresponding to q-value < 0.05).

Prevalence analysis identified significant differences between HC and T1D groups. At the genus level, *Fusarium* (FDR-adjusted *p*-value = 0.019), *Cyberlindnera* (FDR-adjusted *p*-value = 0.028) and *Penicillium* (FDR-adjusted *p*-value = 0.028) were more prevalent in the T1D group. At the species level, *Cyberlindnera jadinii* (FDR-adjusted *p*-value = 0.042) was more prevalent in the T1D group (Figure 4).

To investigate whether fungal alterations were related to glycemic control, participants with T1D were stratified according to HbA1c (<7% *vs*. ≥7%). No significant differences were observed between the two HbA1c groups in alpha diversity, beta diversity or differential fungal abundance.

**Figure 4.**
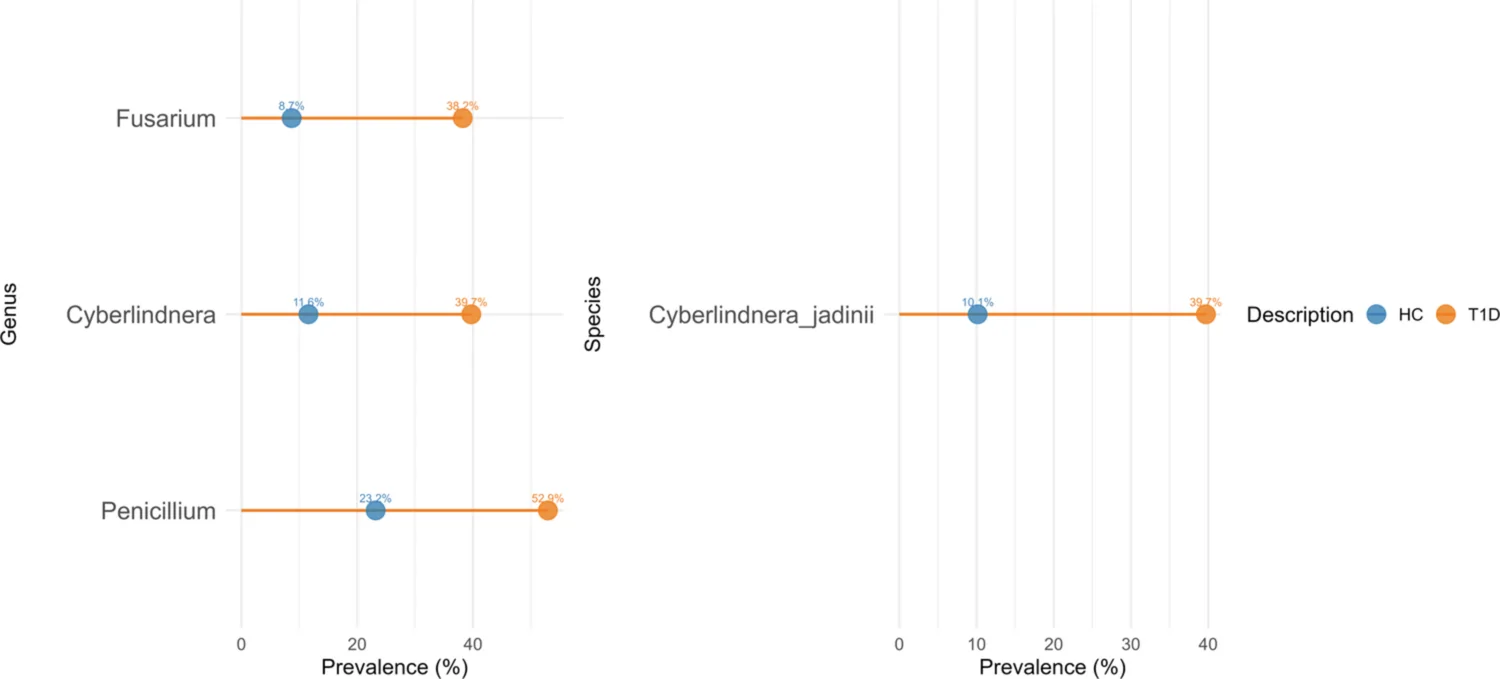
Fungal prevalence differences. Lollipop graph of significantly different prevalent fungi at genus and species levels between healthy controls (HC) and type 1 diabetes (T1D) groups, identified using chi-square tests with FDR adjustment (threshold: FDR *p*-value < 0.05).

### Mycobiome-immunomodulators associations

Descriptive statistics for the 27 measured cytokines are reported in Supplementary Table 1. Cytokine levels were subsequently evaluated in relation to fungal community composition and clinical characteristics.

Multivariable MaAsLin2 analysis was used to investigate associations between fungal taxa and immune mediators after adjustment for age, sex, BoP% and PI%. Several significant positive associations were identified.

At the genus level, *Trametes* was correlated with Macrophage Inflammatory Protein-1β (MIP-1β) (FDR-adjusted *p*-value = 2 × 10^−9^) and Interleukin-10 (FDR-adjusted *p*-value = 0.038); *Cystobasidium* was correlated with Monocyte Chemoattractant Protein-1/Monocyte Chemotactic and Activating Factor (MCP-1/MCAF; FDR-adjusted *p*-value = 2.7 × 10^−6^), and *Trichoderma* was associated with IP-10 (Interferon gamma-induced protein 10; FDR-adjusted *p*-value = 3.1 × 10^−5^) (Figure 5). At the species level, only *Cystobasidium slooffiae* was positively associated with MCP1/MCAF (FDR-adjusted *p*-value = 2.7 × 10^−6^).

The concentrations of the immune mediators identified in the MaAsLin2 analysis were subsequently compared between T1D and HC. Only MIP-1β was significantly increased in the HC group (FDR-adjusted *p*-value = 0.0005) (Figure S1A). Among the fungi significantly correlated with immune modulators, *Cystobasidium slooffiae* and *Trichoderma* were detected exclusively in the T1D group, whereas *Trametes* was more abundant in the HC group. However, these differences were not statistically significant based on the ANCOM-BC analysis (Figure S1B).

**Figure 5.**
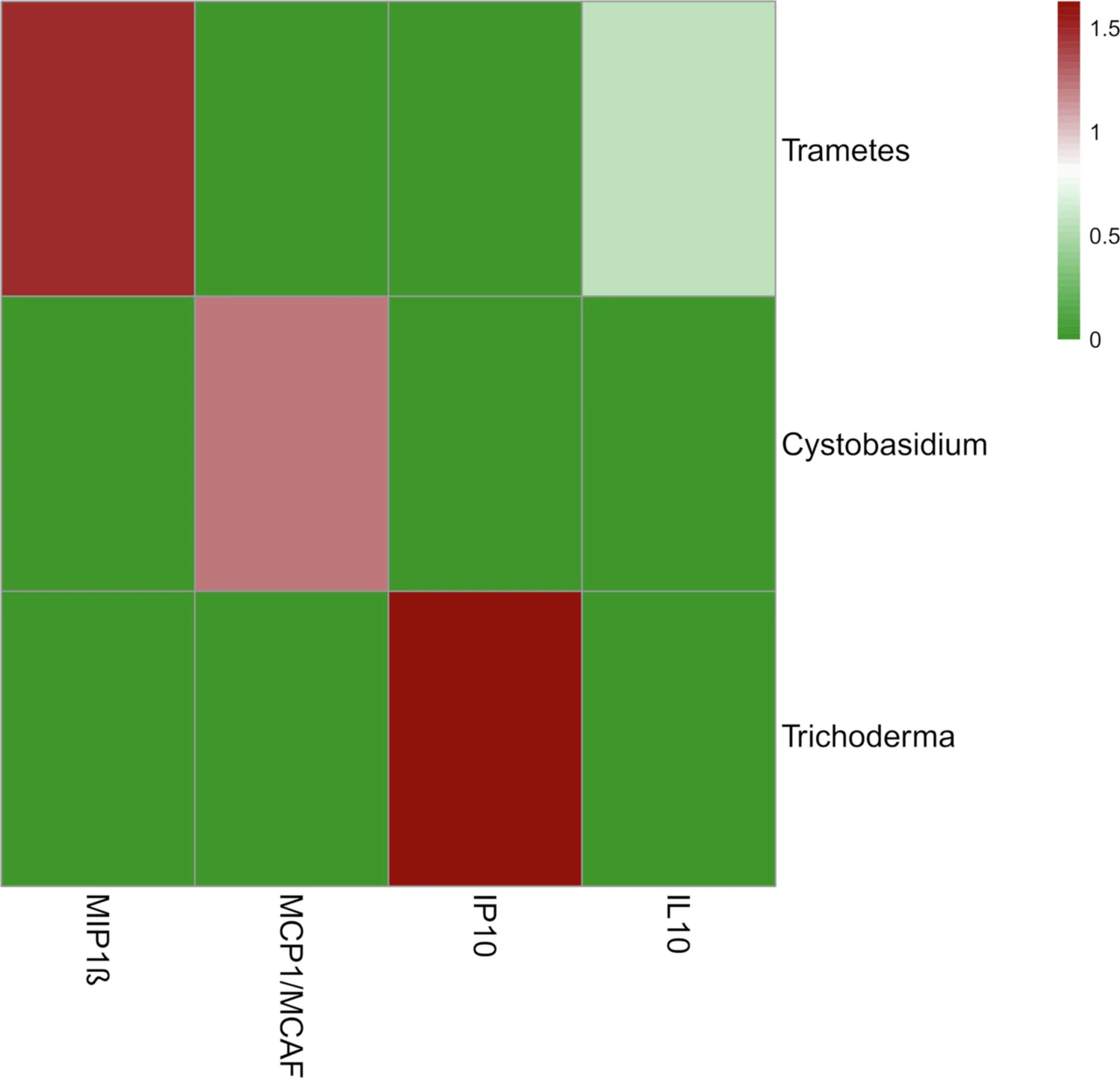
Fungi-cytokine correlations. Heatmap showing significant correlations between fungi and immune modulators, tested by MaAsLin2 models adjusted for age, sex, BoP% and PI%, with FDR-adjusted *p*-values (threshold: FDR *p*-value < 0.05).

To assess whether fungal taxon-cytokine associations differed according to T1D status or glycemic control, we fitted multivariable models including the cytokine concentration, the grouping variable, their interaction and age, sex, PI% and BoP% as covariates. The coefficients reported below correspond to the cytokine-by-group interaction terms. In the HC-versus-T1D analysis, significant differential associations were identified for *Trametes* and G-CSF (*β* = −2.149, SE = 0.170, FDR *p*-value = 7.17 × 10^−21^), *Cystobasidium* and MCP-1/MCAF (*β* = 1.333, SE = 0.230, FDR *p*-value = 2.05 × 10^−5^) and *Trichoderma* and IP-10 (*β* = 1.623, SE = 0.372, FDR *p*-value = 0.00567). The association between *Cystobasidium* and MCP-1/MCAF was also supported at the species level for *Cystobasidium slooffiae* (*β* = 1.301, SE = 0.236, FDR *p*-value = 4.26 × 10^−5^). As HC was the reference group, the negative interaction coefficient for *Trametes* indicates a weaker or more negative G-CSF-*Trametes* association in individuals with T1D, whereas the positive coefficients for *Cystobasidium* and *Trichoderma* indicate stronger or more positive associations in T1D relative to HC. Within the T1D group, a significant interaction was also observed between HbA1c category and IL-5 in relation to *Debaryomyces* (*β* = −3.342, SE = 0.966, FDR *p*-value = 0.04899). With HbA1c < 7% as the reference category, this negative interaction coefficient indicates a weaker or more negative IL-5-*Debaryomyces* association among participants with HbA1c ≥7% (Table 3).

**Table 3.** Differential associations between fungal taxa and immune mediators according to T1D status and glycemic control. Associations were assessed using multivariable linear models including fungal taxon abundance, immune mediator concentration, the relevant grouping variable and the interaction between immune mediator concentration and grouping variable. Models were adjusted for age, sex, PI% and BoP%. For the HC vs. T1D comparison, the interaction term evaluated whether fungal taxon-immune mediator associations differed according to T1D status. Within the T1D group, the interaction term evaluated whether these associations differed between participants with HbA1c < 7% and ≥7%. The *β* coefficients, SEs and FDR *p*-values shown correspond to the interaction terms. Only interaction terms significant after correction for multiple testing are shown.

Effect-modifier comparison	Immune factor	Fungal taxon	β	SE	FDR *p*-value
HC *vs*. T1D	G-CSF	*Trametes*	−2.149	0.170	7.17 × 10^−21^
HC *vs*. T1D	IP-10	*Trichoderma*	1.623	0.372	0.00567
HC *vs*. T1D	MCP-1/MCAF	*Cystobasidium*	1.333	0.230	2.05 × 10^−5^
HC *vs*. T1D	MCP-1/MCAF	*Cystobasidium slooffiae*	1.301	0.236	4.26 × 10^−5^
HbA1c < 7% *vs*. ≥7%	IL-5	*Debaryomyces*	−3.342	0.966	0.0490

### Oral fungi-bacteria networks

After the filtering of mitochondria and unassigned reads, the sequencing output for the bacterial population yielded a total of 3,218,239 sequences (median = 20,041 and IQR = 6,839).

To investigate potential relationships between the bacterial and fungal communities, associations between CLR-transformed relative abundances were first assessed using Spearman's rank correlation. No significant associations were observed between the relative abundances of bacterial and fungal taxa. Similarly, no significant association was observed between bacterial and fungal alpha diversity. At the genus level, a weak negative association was observed between bacterial and fungal Observed ASVs (*r* = −0.16, *p*-value = 0.074), but this did not reach statistical significance (Figure S2).

Correlation analysis based on the presence/absence of bacterial and fungal taxa revealed significant negative associations at the genus level. In particular, *Candida* was negatively correlated with several bacterial genera, *Cutibacterium* (phi = −0.32), *Erysipelotrichaceae*_UCG-006 (phi = −0.32) and *Serratia* (phi = −0.32); *Cyberlindnera* was negatively correlated with *Treponema* (phi = −0.35) *Lautropia* (phi = −0.32), *Corynebacterium* (phi = −0.33) and *Eubacterium* (phi = −0.31); *Cystobasidium* was negatively correlated with *Capnocytophaga* (phi = −0.34); *Malassezia* was negatively correlated with *Shuttleworthia* (phi = −0.49), and *Anaeroglobus* (phi = −0.32) (Figure 6).

**Figure 6.**
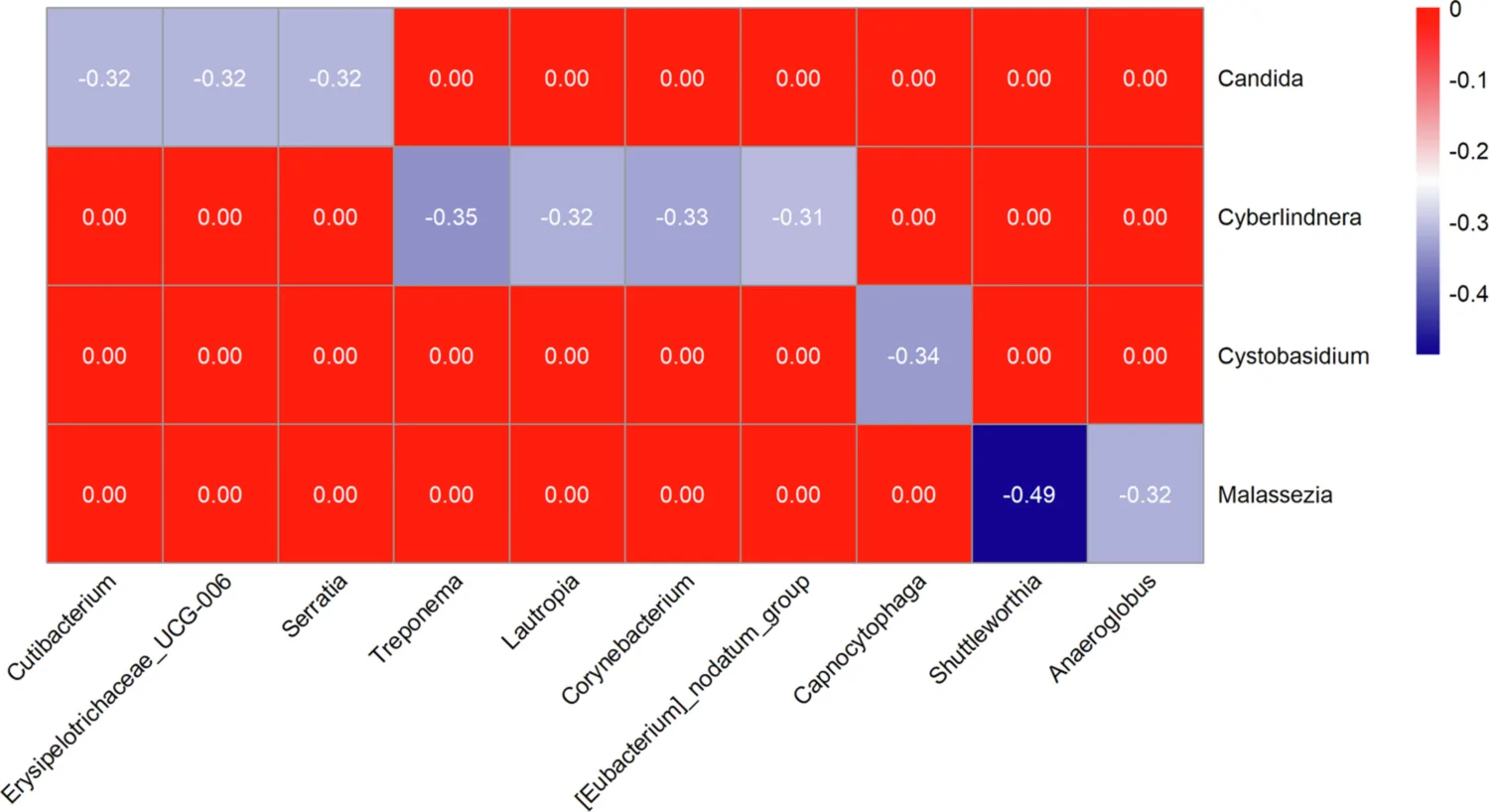
Fungal-bacterial genus associations. Phi coefficient heatmap showing significant correlations (FDR *p*-value < 0.05) between fungal and bacterial genus presence/absence. Negative values (blue cells) indicate negative presence/absence associations; red cells (0) represent no association.

## Discussion

Despite increasing interest in the human microbiome, studies exploring the oral mycobiome, particularly in pediatric populations, remain limited. In this context, the present study addresses this gap by characterizing the oral mycobiome and its associations with the bacterial community in children and young adults with established T1D compared with age-matched HC. Overall, our findings indicate selective differences in the abundance of specific fungal taxa in T1D, rather than broad community-wide restructuring of the oral mycobiome. Importantly, given the cross-sectional design of the study, these findings should be interpreted as microbial features associated with established T1D and do not establish whether these alterations precede, result from or contribute to the development of the disease. Several fungal taxa were enriched in the T1D oral mycobiome, including *Fusarium* spp., *Candida albicans, Candida dubliniensis, Cyberlindnera jadinii* and *Malassezia globosa*. All of them represent common oral colonizers but exert distinct ecological and pathogenic roles. *Fusarium* stands out as an opportunistic pathogen in diabetic wounds, and its enrichment in the T1D oral cavity may therefore be of biological interest, although the present data cannot establish whether this reflects diabetes-related changes in the oral environment, altered host factors or other characteristics of the study population [8,15,16]. In contrast, *Cyberlindnera jadinii* shows probiotic-like anti-inflammatory effects in the gut [17,18]. Its T1D enrichment is noteworthy, but its biological significance in the oral environment remains uncertain. Rather than necessarily representing an adaptive or protective response, this finding may reflect an ecological feature of the oral environment associated with established T1D. Oral-specific studies will be required to determine whether *C. jadinii* has functional effects in this setting.

*Malassezia globosa* has been reported to increase in association with suboptimal glycemic control and metabolic syndrome, conditions that provide a lipid‑rich environment favorable to its growth, as this genus depends on external lipids for survival [19,20]. The increased detection of *M. globosa* in T1D may therefore be compatible with disease-related changes in the oral environment, although the cross-sectional nature of our data does not allow the direction of this association to be established. *Candida dubliniensis* and *C. albicans* are well‑known opportunistic colonizers of the oral cavity, frequently causing thrush in pediatric diabetes [21–24]. In this context, the higher detection of *Candida* in T1D may be consistent with changes in the oral environment associated with diabetes, but the present study cannot determine whether these factors directly account for the observed differences. Other genera, including *Meyerozyma* and *Hanseniaspora*, have also been described as opportunistic fungi in clinical settings [25]; their detection in our cohort should therefore be interpreted cautiously, without assuming that their presence necessarily reflects pathogenic activity [25].

Other fungi, such as *Zygosaccharomyces* and *Vuilleminia,* lack strong human pathogenicity and may represent transient environmental colonizers whose detection could reflect differences in the oral environment associated with established T1D [26,27].

Overall, these findings support the interpretation that T1D is associated with changes in selected fungal taxa rather than a generalized alteration of the oral mycobiome. In our cohort, fungal alpha diversity, beta diversity and differential abundance did not differ significantly between HbA1c% categories (<7% *vs*. ≥7%). This may suggest that the oral mycobiome is influenced more by diabetes-related factors such as immune status, rather than short-term glycemic variation. However, this finding should not be interpreted as evidence that the oral mycobiome is primarily regulated by immune status, as other diabetes-related, oral, behavioral and environmental factors were not comprehensively captured in the present study. Several fungal taxa showed positive associations with immune mediators. *Cystobasidium slooffiae*, an emerging yeast pathogen in immunocompromised conditions, was correlated with MCP-1/MCAF, a key chemokine that regulates migration and infiltration of monocytes/macrophages [28,29]. *Trichoderma* can cause localized and disseminated infections in immunocompromised individuals [30,31], its presence in the oral cavity and its association with pro-inflammatory chemokines such as IP-10 have not been described. Therefore, its role as an immunomodulator in saliva requires confirmation in independent cohorts. To note, both taxa were detected exclusively in the T1D group and were associated with pro-inflammatory mediators. However, the observed correlations cannot establish whether fungal taxa influence immune mediator concentrations, whether immune conditions influence fungal colonization or whether both are related to other characteristics of T1D. Accordingly, these associations should be regarded as hypothesis-generating.

In contrast, *Trametes* was associated with pro-inflammatory MIP-1β but also with the anti-inflammatory IL-10. Although these findings may indicate a complex relationship between specific fungal taxa and immune mediators, they do not demonstrate an anti-inflammatory or immunomodulatory effect of *Trametes* in the oral cavity [32]. To note, *Trametes* was increased in the HC group. Considering that most cytokine concentrations did not significantly differ between HC and T1D, the observed fungus-cytokine correlations may capture a potential relationship between specific fungal taxa and immune activation that is not captured by between-group comparisons alone.

Concerning the prevalence analysis, increased *Cyberlindnera jadinii* in T1D is important in light of its probiotic-like anti-inflammatory effect reported in gut models, although the biological significance of this finding in the oral environment remains uncertain and requires oral-specific validation [17].

In participants with established T1D, several fungal taxa were statistically associated with immune mediators. Of particular note, *Cystobasidium* and *Cystobasidium slooffiae* showed consistent positive associations with MCP-1/MCAF. These findings are compatible with a relationship between specific fungal features and the host immune milieu; however, they do not establish whether fungal abundance influences cytokine levels, whether immune conditions affect fungal abundance or whether both are influenced by other characteristics associated with T1D. *Debaryomyces* was inversely associated with IL-5. Because these analyzes were based on HbA1c categories rather than longitudinal measurements, they should be interpreted as associations with glycemic-control strata rather than evidence that changes in glycemic control drive fungal-immune relationships. The differences between the HC-versus-T1D and HbA1c-stratified analyzes also suggest that the fungal-cytokine associations observed in established T1D cannot be reduced to a single effect of glycemic control as captured by HbA1c category.

Importantly, with the exception of MIP-1β, cytokine levels themselves did not differ significantly between the HC and T1D groups in the overall analysis. Therefore, the taxon-specific associations observed here should not be interpreted as evidence of a generalized inflammatory state in the T1D oral environment. Instead, they suggest that selected components of the oral mycobiome may covary with individual immune mediator profiles.

In the multikingdom analysis, we observed a trend toward a weak inverse association between bacterial and fungal alpha diversity at the genus level that did not reach statistical significance. These patterns may reflect ecological relationships between the two microbial kingdoms, but their underlying mechanisms cannot be determined from the present analysis. We found several negative associations between fungal and bacterial taxa. *Candida* showed moderate inverse correlations with *Cutibacterium*, *Erysipelotrichaceae_*UCG‑006 and *Serratia* [24,33]. Similarly, *Cyberlindnera* was negatively correlated with *Treponema, Lautropia* and *Corynebacterium*, whereas *Cystobasidium* was inversely correlated with *Capnocytophaga*, indicating that the expansion of specific fungal taxa in T1D may coincide with the depletion of bacterial groups linked to periodontal health and mucosal homeostasis [34]. The strongest negative correlation was observed between *Malassezia* and *Shuttleworthia* [35]. However, these statistical associations should not be interpreted as evidence of direct competition, antagonism, niche exclusion or functional interaction. Detection-based co-occurrence analyzes can be affected by sparsity and by the compositional nature of sequencing data, and negative associations may arise through direct or indirect ecological relationships as well as through other unmeasured factors.

Several limitations should be considered. The broad age range (6-21 years) spans distinct developmental stages, and residual confounding by age or pubertal status cannot be excluded. The 14-day washout for antibiotics, antimycotics and anti-inflammatory drugs was a pragmatic compromise in a pediatric cohort, but longer-lasting effects on the oral microbiome may persist. Potential selection bias related to control recruitment from the Emergency Department also cannot be excluded. Although oral hygiene was partly captured by PI% and BoP%, information on other potentially relevant factors, including recent dental care, xerostomia/salivary flow, diet, socioeconomic factors, pubertal stage and some diabetes-specific characteristics, was incomplete or unavailable. We therefore used a parsimonious set of a priori confounders (age, sex, PI% and BoP%) to limit overfitting. Residual confounding and compositionality-related biases inherent to sequencing data remain possible.

The study also has important strengths, including the combined characterization of the bacterial microbiome and mycobiome, detailed clinical and oral phenotyping, well-characterized controls from the same geographic area and integration of glycemic control into the analyzes. These features provide a comprehensive characterization of the oral mycobiome in a clinically relevant pediatric T1D population and identify associations for future longitudinal and functional investigation.

## Conclusions

In conclusion, the present study identifies selective differences in the oral mycobiome of children and young adults with established T1D, including enrichment of specific fungal taxa such as *Candida* spp., *Cyberlindnera jadinii*, *Fusarium* spp. and *Malassezia globosa*. These findings are not accompanied by broad differences in community diversity and therefore are more appropriately interpreted as taxon-specific compositional shifts rather than generalized oral mycobiome dysbiosis. Associations between selected fungal taxa and immune mediators further suggest potential biological relevance, although their directionality and functional consequences remain to be established. The multikingdom analysis additionally identified inverse associations between selected fungal and bacterial taxa. These findings provide a basis for further investigation of the relationships between the two microbial kingdoms but should not be interpreted as evidence of direct competition, antagonism or other specific ecological mechanisms. More broadly, because the study is cross-sectional and includes participants with prevalent T1D, the observed microbial features cannot be assigned a causal role in diabetes development or systemic autoimmunity. Taken together, these results support further prospective investigation of the oral mycobiome in T1D, particularly through longitudinal studies integrating microbial, metabolic, immunological and oral-health measures, irrespective of glycemic control [36]. Such studies will be necessary to determine the stability, temporal direction and functional relevance of the observed microbial associations and to assess whether specific oral mycobiome signatures may ultimately have value as markers of disease-associated oral ecological changes or oral complications.

## Supplementary Material

Supplementary.docx Supplementary.docx

## Data Availability

The datasets presented in this study can be found in online repositories. The names of the repository/repositories and accession number(s) can be found below: http://www.ncbi.nlm.nih.gov/bioproject/1456630; PRJNA1456630.

## References

[1] Jacobsen LM , Schatz DA . Type 1 diabetes: a review. J Am Med Assoc. 2026;335:1070–1083. doi: 10.1001/jama.2026.0048 41697686

[2] Lee HS , Hwang JS . Genetic aspects of type 1 diabetes. Ann Pediatr Endocrinol Metab. 2019;24:143–148. doi: 10.6065/apem.2019.24.3.143 PMC6790877 31607106

[3] Tang R , Shi M , Ji X , et al. Integrative oral and gut microbiome profiling highlights microbial correlates of complications in type 1 diabetes: a cross-sectional analysis. Cardiovasc Diabetol. 2025;24:461. doi: 10.1186/s12933-025-03012-z PMC12729026 41444596

[4] Aldegheri L , Catamo E , Zanotta N , et al. Alterations of oral microbiota and cytokines profile in children and young adults with type 1 diabetes. Front Endocrinol (Lausanne). 2025;16:1629185. doi: 10.3389/fendo.2025.1629185 PMC12520906 41103647

[5] Deo PN , Deshmukh R . Oral microbiome: unveiling the fundamentals. J Oral Maxillofac Pathol. 2019;23:122–128. doi: 10.4103/jomfp.JOMFP_304_18 PMC6503789 31110428

[6] Xu H , Dongari-Bagtzoglou A . Shaping the oral mycobiota: interactions of opportunistic fungi with oral bacteria and the host. Curr Opin Microbiol. 2015;26:65–70. doi: 10.1016/j.mib.2015.06.002 PMC4577367 26100661

[7] Xiang Z , Liu Y . The pediatric oral mycobiome: a comprehensive review of its role in health and disease. Front Cell Infect Microbiol. 2026;16:1711789. doi: 10.3389/fcimb.2026.1711789 PMC12891119 41684946

[8] Ghannoum MA , Jurevic RJ , Mukherjee PK , et al. Characterization of the oral fungal microbiome (Mycobiome) in healthy individuals. PLoS Pathog. 2010;6:e1000713. doi: 10.1371/journal.ppat.1000713 PMC2795202 20072605

[9] Vila T , Sultan AS , Montelongo-Jauregui D , et al. Oral candidiasis: a disease of opportunity. J Fungi (Basel). 2020;6:15. doi: 10.3390/jof6010015 PMC7151112 31963180

[10] Guo ZL , Cui MW , Dong YL , et al. The oral microbiome as a regulatory hub for systemic health: a systematic review of mechanistic links and clinical implications. J Oral Microbiol. 2026;18:2635233. doi: 10.1080/20002297.2026.2635233 PMC12958392 41788296

[11] Defta CL , Albu C-C , Albu Ş-D , et al. Oral mycobiota: a narrative review. Dent J (Basel). 2024;12:115. doi: 10.3390/dj12040115 PMC11049401 38668027

[12] Honkanen J , Vuorela A , Muthas D , et al. Fungal dysbiosis and intestinal inflammation in children with beta-cell autoimmunity. Front Immunol. 2020;11:468. doi: 10.3389/fimmu.2020.00468 PMC7103650 32265922

[13] de Bock M , Codner E , Craig ME , et al. ISPAD clinical practice consensus guidelines 2022: glycemic targets and glucose monitoring for children, adolescents, and young people with diabetes. Pediatr Diabetes. 2022;23:1270–1276. doi: 10.1111/pedi.13455 PMC10107615 36537523

[14] Manerkar M , de Jesus VC , Mittermuller B-A , et al. Effects of silver diamine fluoride on oral bacteriome and mycobiome: a randomized clinical trial. BMC Oral Health. 2025;25:1643. doi: 10.1186/s12903-025-06986-0 PMC12539150 41120980

[15] João AL , Lencastre A , Dutra E , et al. Fusarium spp.—An emerging pathogen in chronic diabetic ulcer: case report and review of the literature. Int J Low Extrem Wounds. 2021;20:67–72. doi: 10.1177/1534734619879030 31722577

[16] Pai R , Boloor R , Shreevidya K , et al. Fusarium solani: an emerging fungus in chronic diabetic ulcer. J Lab Physicians. 2010;2:37–39. doi: 10.4103/0974-2727.66710 PMC3147084 21814405

[17] Hugot C , Poirier M , Spatz M , et al. Cyberlindnera jadinii and kluyveromyces lactis, two fungi used in food processes, have potential probiotic effects on gut inflammation. mSystems. 2023;8:e0084123. doi: 10.1128/msystems.00841-23 PMC10734524 37882535

[18] Mohamed N , Litlekalsøy J , Ahmed IA , et al. Analysis of salivary mycobiome in a cohort of oral squamous cell carcinoma patients from Sudan identifies higher salivary carriage of malassezia as an independent and favorable predictor of overall survival. Front Cell Infect Microbiol. 2021;11:673465. doi: 10.3389/fcimb.2021.673465 PMC8547610 34712619

[19] Celis Ramírez AM , Amézquita A , Cardona Jaramillo JEC , et al. Analysis of malassezia lipidome disclosed differences among the species and reveals presence of unusual yeast lipids. Front Cell Infect Microbiol. 2020;10:338. doi: 10.3389/fcimb.2020.00338 PMC7374198 32760678

[20] Vergès B . Lipid disorders in type 1 diabetes. Diabetes & Metabolism. 2009;35:353–360. doi: 10.1016/j.diabet.2009.04.004 19733492

[21] Mohammadi F , Javaheri MR , Nekoeian S , et al. Identification of candida species in the oral cavity of diabetic patients. Curr Med Mycol. 2016;2:1–7. doi: 10.18869/acadpub.cmm.2.2.4 PMC5490298 28681013

[22] Naeem M . Oral candidiasis in diabetes patients. Wasit J Pure Sci. 2024;3:151–155. doi: 10.31185/wjps.525

[23] Nageeb WM , Abo-Elsoud AAE , Amin MK , et al. Comparative evaluation of oral microbiologic profile in children with type 1 diabetes mellitus versus healthy controls and its relation to oral health status. BMC Oral Health. 2025;25:698. doi: 10.1186/s12903-025-06013-2 PMC12065165 40346500

[24] Rodrigues CF , Rodrigues ME , Henriques M . Candida sp. Infections in patients with diabetes mellitus. J Clin Med. 2019;8:76. doi: 10.3390/jcm8010076 PMC6352194 30634716

[25] Pinto TN , Oliveira LMA , da Costa GL , et al. Detection of hanseniaspora opuntiae in anovaginal samples of pregnant women in rio de janeiro, Brazil—a case report. Front Cell Infect Microbiol. 2024;14:1394663. doi: 10.3389/fcimb.2024.1394663 PMC11169740 38873099

[26] Ghobad-Nejhad M , Nilsson RH , Hallenberg N . Phylogeny and taxonomy of the genus vuilleminia (Basidiomycota) based on molecular and morphological evidence, with new insights into corticiales. Taxon. 2010;59:1519–1534. doi: 10.1002/tax.595016

[27] Wang D , Zhang M , Huang J , et al. Zygosaccharomyces rouxii combats salt stress by maintaining cell membrane structure and functionality. J Microbiol Biotechnol. 2020;30:62–70. doi: 10.4014/jmb.1904.04006 PMC9728352 31635442

[28] Deshmane SL , Kremlev S , Amini S , et al. Monocyte chemoattractant protein-1 (MCP-1): an overview. J Interferon Cytokine Res. 2009;29:313–326. doi: 10.1089/jir.2008.0027 PMC2755091 19441883

[29] Huang J , Guo L , Zhang G , et al. First report of cystobasidium slooffiae in human wounds from China: molecular identification and clinical insights. Infect Drug Resist. 2025;18:5877–5886. doi: 10.2147/IDR.S551470 PMC12619543 41246004

[30] Mendes-Frias A , Viana da Costa A , Salazar F , et al. Effect of type 1 diabetes on the inflammatory response in periodontal disease. Front Immunol. 2025;16:1683219. doi: 10.3389/fimmu.2025.1683219 PMC12631499 41280935

[31] Sal E , Stemler J , Salmanton-García J , et al. Invasive trichoderma spp. Infections: clinical presentation and outcome of cases from the literature and the FungiScope® registry. J Antimicrob Chemother. 2022;77:2850–2858. doi: 10.1093/jac/dkac235 PMC9525085 35929089

[32] Benson KF , Stamets P , Davis R , et al. The mycelium of the trametes versicolor (Turkey tail) mushroom and its fermented substrate each show potent and complementary immune activating properties in vitro. BMC Complement Altern Med. 2019;19:342. doi: 10.1186/s12906-019-2681-7 PMC6889544 31791317

[33] Matsha TE , Prince Y , Davids S , et al. Oral microbiome signatures in diabetes mellitus and periodontal disease. J Dent Res. 2020;99:658–665. doi: 10.1177/0022034520913818 32298191

[34] Rajasekaran JJ , Krishnamurthy HK , Bosco J , et al. Oral microbiome: a review of its impact on oral and systemic health. Microorganisms. 2024;12:1797. doi: 10.3390/microorganisms12091797 PMC11434369 39338471

[35] Wang Y , Wang S , Wu C , et al. Oral microbiome alterations associated with early childhood caries highlight the importance of carbohydrate metabolic activities. mSystems. 2019;4:e00450–e00419. doi: 10.1128/mSystems.00450-19 PMC6832018 31690590

[36] Catamo E , Tornese G , Navarra C , et al. Oral health and salivary inflammatory markers in children and adolescents with type 1 diabetes: a cross-sectional study. J Endocrinol Invest. 2026;49:1403–1412. doi: 10.1007/s40618-026-02808-4 PMC13219055 41505053

