# Supplementary material for: Oral mycobiome composition and fungi-bacteria associations in children and young adults with type 1 diabetes
Source: J Oral Microbiol. 2026 Oct 29;18(1):2750003. doi: 10.1080/20002297.2026.2750003 (PMC13721826; doi:10.1080/20002297.2026.2750003)
Supplement: Supplementary file 1 [file ZJOM_A_2750003_SM5913.docx]

***Supplementary Table 1.*** *Cytokine concentrations (pg/mL) in healthy controls (HC) and subjects with type 1 diabetes (T1D). Values are expressed as median (IQR).*

| **Cytokine** | **HC** | **T1D** | **T1D, HbA1c<7%** | **T1D, HbA1c≥7%** |
| --- | --- | --- | --- | --- |
| IL-1β | 25.37  [12.35-54.03] | 43.14  [25.71-137.59] | 28.46  [21.93-62.45] | 75.94  [29.09-166.22] |
| IL-1ra | 5861.18  [3707.58-8654.68] | 7553.49  [4108.99-12378.29] | 4742.59  [3112.39-8349.30] | 9762.25  [5948.74-15570.89] |
| IL-2 | 2.57  [2.08-4.25] | 4.39  [2.57-5.98] | 2.57  [2.31-4.31] | 5.51  [3.54-7.36] |
| IL-4 | 0.83  [0.59-1.04] | 0.64  [0.27-1.04] | 0.43  [0.23-0.97] | 0.75  [0.39-1.14] |
| IL-5 | 35.64  [22.01-59.02] | 12.11  [12.11-46.92] | 12.11  [12.11-46.34] | 22.65  [12.11-46.92] |
| IL-6 | 12.16  [6.47-20.35] | 9.21  [5.13-16.25] | 6.58  [3.92-12.16] | 13.96  [6.03-18.95] |
| IL-7 | 6.87  [4.10-9.86] | 7.75  [4.57-12.86] | 6.43  [3.47-13.52] | 8.18  [5.75-12.86] |
| IL-8 | 854.70  [484.09-1737.61] | 745.14  [369.25-1937.62] | 423.65  [245.17-901.39] | 1153.09  [472.88-2241.45] |
| IL-9 | 24.20  [17.67-30.20] | 18.78  [8.82-28.79] | 15.09  [5.54-19.51] | 22.85  [14.13-33.42] |
| IL-10 | 3.03  [1.55-4.90] | 3.36  [1.39-5.78] | 2.38  [1.22-5.02] | 3.68  [1.55-7.06] |
| IL-12(p70) | 2.70  [2.15-7.03] | 2.15  [2.15-6.21] | 2.15  [2.15-3.74] | 4.08  [2.15-7.42] |
| IL-13 | 0.37  [0.27-0.56] | 0.37  [0.17-0.56] | 0.34  [0.17-0.56] | 0.42  [0.17-0.58] |
| IL-15 | 48.02  [22.32-126.92] | 22.32  [22.32-114.67] | 22.32  [22.32-59.44] | 55.44  [22.32-127.53] |
| IL-17 | 5.72  [4.28-7.21] | 5.54  [2.81-9.02] | 4.10  [2.35-6.80] | 6.26  [3.54-9.73] |
| Eotaxin | 1.10  [0.79-1.37] | 0.63  [0.23-1.16] | 0.33  [0.19-1.29] | 0.74  [0.42-0.94] |
| FGF basic | 8.77  [6.96-11.46] | 12.11  [7.67-15.69] | 7.89  [5.43-12.20] | 13.18  [10.60-17.41] |
| G-CSF | 162.50  [77.31-272.98] | 175.35  [114.92-270.73] | 132.95  [100.22-243.27] | 210.00  [143.02-270.73] |
| GM-CSF | 1.69  [0.98-2.15] | 0.73  [0.39-2.02] | 0.39  [0.39-1.52] | 0.94  [0.54-2.05] |
| IFN-γ | 184.12  [157.68-208.69] | 16.29  [12.29-170.86] | 13.43  [11.55-179.56] | 18.14  [13.77-87.64] |
| IP-10 | 81.45  [21.33-343.44] | 186.06  [41.96-493.77] | 160.37  [56.89-504.49] | 241.79  [39.62-465.91] |
| MCP-1(MCAF) | 120.96  [60.02-204.47] | 157.36  [87.98-240.48] | 141.63  [52.72-181.60] | 160.78  [96.78-259.48] |
| MIP-1α | 0.84  [0.42-1.97] | 0.74  [0.33-1.31] | 0.54  [0.21-0.93] | 0.87  [0.59-1.39] |
| PDGF-bb | 4.05  [4.05-4.05] | 4.05  [2.38-7.23] | 4.05  [1.67-4.05] | 4.07  [3.67-7.77] |
| MIP-1β | 11.78  [9.28-15.69] | 6.24  [2.58-11.28] | 5.27  [1.44-9.50] | 7.04  [4.01-11.62] |
| RANTES | 4.63  [1.34-10.84] | 4.78  [1.62-9.80] | 1.90  [1.34-5.52] | 5.91  [4.48-10.78] |
| TNF-α | 36.10  [16.92-69.37] | 38.86  [17.29-63.44] | 19.84  [13.02-50.74] | 46.37  [26.27-90.39] |
| VEGF | 394.29  [285.71-558.99] | 645.26  [412.36-891.25] | 497.28  [255.27-717.14] | 750.63  [485.91-914.50] |

***Figure S1. Cytokine-associated fungal profiles.*** ***A)*** *Bar plot showing the concentrations (pg/mL) of immune modulators significantly correlated with fungi in healthy controls (HC) and type 1 diabetes (T1D) groups. Except for MIP-1β, these cytokines were not different between groups according to the Wilcoxon test.* **B)** *Bar plot showing the mean relative abundances of the significantly correlated fungi with immune modulators in HC and T1D groups. These fungi were not significantly different between groups according to the ANCOM-BC test. Abbreviations: ns: not significant, *** = FDR p-value < 0.001.*

***Figure S2. Bacterial-fungal diversity correlation.*** *Spearman rank correlation between bacterial and fungal alpha diversity. No significant correlation was detected.*
